# Perinatal outcomes in neonates referred to social services in a tertiary neonatal intensive care unit from a resource-limited setting: a five-year study

**DOI:** 10.3389/fped.2025.1760975

**Published:** 2026-01-22

**Authors:** Nazan Neslihan Dogan Kocabıyık, Ozgul Salihoglu

**Affiliations:** Bakırkoy Dr. Sadi Konuk Training Research Center, Department of Pediatrics, Division of Neonatology, University of Health Sciences, Istanbul, Türkiye

**Keywords:** infant, newborn, intensive care units, neonatal, prenatal care, social determinants of health, social services

## Abstract

**Background:**

Beyond medical complexity, social determinants of health and social vulnerability have emerged as factors shaping perinatal and neonatal outcomes among socially vulnerable families in neonatal intensive care units. However, data integrating maternal, neonatal, and social risk factors among infants requiring social-service referral during neonatal intensive care unit (NICU) hospitalization remain limited, especially in resource-limited settings.

**Study design:**

A retrospective cohort study was conducted including all neonates who received a formal social-service consultation between January 2020 and December 2024, and a comparison group of infants hospitalized without referral.

**Methods:**

Data were extracted from electronic medical records and social-service notes. Maternal variables included age, marital status, antenatal visit frequency, prenatal testing, smoking or substance use, hematologic parameters, and antenatal corticosteroid administration. Neonatal variables included birth weight, gestational age, APGAR scores, NICU diagnoses, prematurity-related morbidities, respiratory support, thyroid function tests, hearing-screening results, and hospitalization duration. Social-service notifications were categorized as legal/judicial reasons, parental psychosocial or functional challenges, parental care risks, and socioeconomic vulnerabilities. Group comparisons used *t*-tests, Mann–Whitney *U* tests, and *χ*^2^/Fisher's exact tests. Logistic regression identified independent predictors of referral.

**Results:**

A total of 193 neonates were assessed. Referred infants (*n* = 96) were born to younger mothers with significantly higher rates of adolescent pregnancy, unmarried status, inadequate antenatal care, lower maternal hemoglobin levels, and higher smoking/substance use. Referred infants had lower 5-min APGAR scores and higher rates of low birth weight, neurological diagnoses, bronchopulmonary dysplasia, abnormal thyroid function, prolonged hospitalization, and bilateral or unilateral hearing-screen failure. Mortality was significantly higher in the referred group. In multivariate analysis, lack of legal marriage (OR: 0.05), absence of antenatal care (OR: 0.12), lower maternal hemoglobin (OR: 0.41), lower neonatal TSH levels (OR: 0.75), and longer hospitalization (OR: 1.07) remained independent predictors of social-service referral. Non-Turkish nationality was significant in univariate analysis but not in the adjusted model.

**Conclusion:**

Infants referred to social services in the NICU represent a distinctly vulnerable population characterized by inadequate maternal antenatal care, unmarried status, maternal anemia, and substance exposure. Integrating early social-risk screening into routine antenatal care and strengthening multidisciplinary perinatal–social collaboration may improve outcomes in high-risk families.

## What is known?

- Maternal social vulnerability—particularly adolescent pregnancy, unmarried status, inadequate antenatal care, and substance use—is strongly associated with adverse perinatal outcomes.
- Non-citizen mothers experience structural barriers to healthcare access, which can contribute to delayed or insufficient prenatal care and higher neonatal risk.
- Social-service referrals in the NICU commonly arise from concerns about caregiving capacity, safety, or psychosocial instability, yet standardized risk-assessment frameworks remain limited.

## What is new?

- This study highlights social-service referral in the NICU as a marker of combined medical and social vulnerability rather than an isolated social or clinical event.
- It emphasizes the role of social-service involvement as a family-centered, supportive process rather than a separation-based intervention, aligning with contemporary attachment-preserving care principles.
- The findings draw attention to potential under-recognition of social risk among neonates not referred to social services, underscoring the need for standardized social-risk screening in NICU practice.
- By integrating maternal, neonatal, and social determinants within a high-acuity neonatal setting, this study contributes novel insight from a resource-limited context, where such data remain scarce.
- Findings underscore the need for integrated perinatal–social care models incorporating early social-risk screening and structured NICU discharge planning.

## Introduction

Neonatal outcomes in intensive care settings are influenced not only by clinical conditions but also by social determinants of health. The World Health Organization identifies these determinants as major contributors to perinatal health disparities and key drivers of inequities in maternal–infant outcomes (1). Adverse social conditions—including younger maternal age, unmarried status, low educational attainment, inadequate antenatal care, and tobacco or substance use—have consistently been associated with preterm birth, low birth weight, and increased neonatal morbidity (2–5). These pathways extend beyond biological vulnerabilities. Limited parental caregiving capacity, unstable family structures, maternal psychosocial challenges, and household safety concerns often necessitate postnatal social-service intervention (6). Consequently, close integration between clinical teams and social-service professionals is essential to ensure safe discharge planning and provide adequate support for socially vulnerable newborns (7, 8). Although the role of social workers in adult critical care has been documented (7, 8), data on neonates formally referred to hospital social services remain limited. Recent studies highlight that maternal social determinants significantly influence major neonatal morbidities, such as hypoxic–ischemic encephalopathy and other adverse perinatal outcomes (2, 3). These findings emphasize the need for structured evaluation of social risk factors within NICUs. This study aims to systematically assess the demographic, maternal, and clinical characteristics of neonates referred to social services in a tertiary NICU over a five-year period. By concurrently examining medical and social risk domains, the present study addresses an important gap in the literature and contributes novel evidence on the intersection of social vulnerability and neonatal outcomes.

## Materials and methods

### Study design

This retrospective, descriptive, and analytical observational study was conducted in the neonatal intensive care unit (NICU) of a tertiary training and research hospital between January 2020 and December 2024. The study adheres to the STROBE (Strengthening the Reporting of Observational Studies in Epidemiology) guidelines.

### Ethical approval

The research protocol was approved by the local institutional ethics committee (Approval No. 2025-07-12; Date: April 9, 2025). Owing to the retrospective design and complete anonymization of data, the requirement for informed consent was waived. All procedures were performed in accordance with the Declaration of Helsinki.

### Study population

No sampling method was used. All neonates who received a formal social-service referral (consultation request) during the study period constituted the referral group (*n* = 96). A comparison group (non-referral group) consisted of 97 neonates hospitalized in the same NICU during the same years without a social-service referral. The total study cohort comprised 193 infants.

### Data collection and variables

Data were extracted retrospectively from electronic health records and documented social-service referral and consultation notes.

### Maternal & neonatal variables

Maternal variables included maternal age, marital status, gestational age, presence and frequency of antenatal visits, prenatal testing, mode of delivery, hemoglobin and hematocrit values, TSH and FT4 levels, maternal history of chronic disease, smoking and substance use, and administration of antenatal corticosteroids. Neonatal variables included birth weight, gestational age, sex, APGAR scores, congenital anomalies, NICU admission diagnoses, prematurity-related morbidities (RDS, BPD, NEC, IVH, PDA, sepsis, pneumothorax, ROP), respiratory support, surfactant use, results of newborn hearing screening, age at admission, length of hospital stay, discharge status, and discharge destination.

### Social-service notification categories

Notifications were classified into four mutually exclusive categories based on standardized hospital codes:

1. Legal/Judicial reasons
2. Parental Psychosocial and Functional Challenges
3. Parental Care and Behavioral Risks
4. Socioeconomic/Structural Vulnerabilities

### Definitions

All variables were classified according to standardized definitions widely used in perinatal and neonatal research:

- **Adolescent pregnancy**: Maternal age between 10 and 19 years (9).
- **Inadequate antenatal care**: Defined as fewer than four antenatal visits during pregnancy, consistent with thresholds commonly applied in studies assessing antenatal care adequacy and maternal social deprivation (10–13).
- **Intermediate antenatal care:** Defined as five to seven antenatal visits during pregnancy, representing a partially adequate level of antenatal care utilization (10–13).
- **Adequate antenatal care**: Eight or more antenatal visits (10–13).
- **Low birth weight (LBW)**: Birth weight <2500 g.
- **Preterm birth**: Gestational age <37 weeks.
- **Neonatal morbidity:** Neonatal morbidity was defined as the presence of at least one major complication commonly reported as a marker of illness severity in NICU populations, including respiratory distress syndrome, bronchopulmonary dysplasia, necrotizing enterocolitis, intraventricular hemorrhage, patent ductus arteriosus, sepsis, pneumothorax, and retinopathy of prematurity. Although the study population included both preterm and term infants, these conditions were selected because they are among the most frequently documented and clinically relevant morbidities across NICU populations. Other condition-specific morbidities, such as surgery-related complications or hypoxic–ischemic encephalopathy, were not included in this composite outcome to avoid heterogeneity and preserve methodological consistency.
- **Substance use**: Documented use of illicit drugs or alcohol during pregnancy.
- Chronic maternal disease: Pre-existing medical conditions such as hypertension, diabetes, or epilepsy diagnosed before pregnancy.

### Statistical analysis

Statistical analyses were performed using the Jamovi statistical software (version 2.3.28; Sydney, Australia). The distribution of continuous variables was assessed using the Shapiro–Wilk test. Normally distributed variables were summarized as mean ± standard deviation, and non-normally distributed variables as median [minimum–maximum].

For comparisons between groups:

- Parametric continuous variables: Independent Samples *t*-test
- Non-parametric continuous variables: Mann–Whitney *U* test
- Categorical variables: Pearson's Chi-square test
- 2 × 2 tables with expected cell counts <5: Fisher's Exact test
- Multi-category R × C tables: Fisher–Freeman–Halton test

Missing data were addressed using univariate median imputation, based on the distribution of each variable. To identify factors associated with social-service referral, univariate and multivariate logistic regression analyses were conducted. Results of logistic regression were reported as Odds Ratios (ORs) with 95% confidence intervals (CIs) and corresponding *p*-values. A two-tailed *p*-value <0.05 was considered statistically significant. Given the retrospective and observational nature of the study, no formal *a priori* sample size calculation was performed. Multivariable logistic regression was conducted in accordance with commonly accepted events-per-variable considerations to minimize overfitting.

## Results

A total of 193 neonates were included in the analysis, of whom 96 were referred to social services (referral group) and 97 were not (non-referral group). Maternal and obstetric characteristics differed significantly between groups (Table 1). Mothers in the referral group were more frequently of non-Turkish nationality, were younger, and had a substantially higher rate of adolescent pregnancy. Rates of being legally married and attending antenatal visits were markedly lower in this group. Hemoglobin and hematocrit levels were significantly reduced, whereas smoking and substance use during pregnancy were considerably more common among referred mothers. Prenatal testing was also performed far less frequently in the referral group compared with the non-referral group. Neonatal characteristics and clinical outcomes are summarized in Table 2. Infants in the referral group had significantly lower 5-min APGAR scores and higher rates of low birth weight. Although overall NICU admission rates were similar, diagnostic distributions differed between groups, with neurological problems and prematurity-related conditions occurring more frequently among referred infants. Bronchopulmonary dysplasia was also significantly more common in this group. Laboratory findings are presented in Table 3. Thyroid function tests demonstrated significantly lower TSH and free T4 levels in the referral group. Length of hospitalization was substantially longer among referred neonates. Hearing-screening outcomes differed significantly between groups, with higher rates of unilateral and bilateral failures in the referral group. Social-service notification categories are shown in Table 4. The most common reason for referral was parental care and behavioral risks, followed by parental psychosocial and functional challenges, legal/judicial issues, and socioeconomic/structural vulnerabilities. Mortality and discharge characteristics are presented in Table 5. Mortality was significantly higher among referred neonates. Only 37.5% of infants in this group were discharged home, whereas over half were transferred to social-service institutions. In contrast, nearly all infants in the non-referral group were discharged home. Factors associated with social-service referral are listed in Table 6. In univariate analyses, nationality, marital status, antenatal visit status, maternal hemoglobin, birth weight, 5-min APGAR score, neurological problems, TSH level, hearing-screening results, and length of stay were significantly associated with referral. In the multivariate model, lack of legal marriage, absence of antenatal visits, lower maternal hemoglobin, lower neonatal TSH, and prolonged hospitalization were identified as independent predictors of social-service referral.

**Table 1:** Maternal characteristics by social-service referral Status.

Variables	Social service referral		*p*
	No (*n* = 97)	Yes (*n* = 96)	
Nationality^‡^			
Turkish	83 (85.6)	57 (59.4)	**<0.001** *
Others	14 (14.4)	39 (40.6)	
Maternal Age^†^ (years)	29.9 ± 6.1	26.7 ± 7.8	**0.002** ***
Adolescent pregnancy^‡^, yes	2 (2.1)	24 (26.1)	**<0.001** *
Gravidity^§^	2.0 [1.0–10.0]	2.0 [1.0–7.0]	0.206**
Parity^§^	2.0 [1.0–9.0]	2.0 [1.0–5.0]	0.127**
Legally Married^‡^, yes	87 (89.7)	33 (35.9)	**<0.001** *
Received Prenatal Visits^‡^, yes	77 (79.4)	24 (26.1)	**<0.001** *
Prenatal Visits^‡^			
Irregular	47 (61.0)	18 (75.0)	0.316*
Regular	30 (39.0)	6 (25.0)	
Gestational Complications^‡^, yes	31 (33.7)	21 (52.5)	0.066*
OGTT performed^‡^, yes	15 (15.5)	4 (4.3)	**0.022** *
Prenatal Testing^‡^, yes	31 (32.0)	4 (4.3)	**<0.001** *
Chronic disease history^‡^, yes	23 (23.7)	13 (14.1)	0.136*
Maternal hemoglobin^†^ (g/dL)	11.0 ± 1.5	10.3 ± 1.2	**<0.001** ***
Maternal hematocrit^†^	33.8 ± 3.9	31.4 ± 3.1	**<0.001** ***
Smoking during pregnancy^‡^, yes	19 (19.6)	53 (57.6)	**<0.001** *
Substance use during pregnancy^‡^, yes	0 (0.0)	28 (30.4)	**<0.001** *
Antenatal steroids^‡^, yes	8 (8.2)	4 (4.3)	0.423*
Multiple Gestation^‡^, yes	1 (1.0)	3 (3.2)	0.366*
Maternal Serology^‡^			
Normal	97 (100.0)	85 (93.4)	**0.009** *
AntiHCV+	0 (0.0)	5 (5.5)	
HBsAg+	0 (0.0)	1 (1.1)	
Preterm Delivery^‡^, yes	34 (35.1)	38 (39.6)	0.616*
Mode of Delivery^‡^			
Normal vaginal delivery	20 (20.6)	35 (37.6)	**0.015** *
Cesarean section (c/s)	77 (79.4)	58 (62.4)	

AntiHCV, antibodies against the hepatitis C virus; HBsAg, hepatitis B surface antigen; OGTT, oral glucose tolerance test.

Bold values indicate statistically significant results (*p* < 0.05).

* Pearson Chi-Square, Fisher's Exact/Fisher Freeman Halton test.

** Mann–Whitney *U* test.

*** Independent samples *t*-test.

† Mean ± standard deviation.

‡ *n* (%).

§ Median [Min.–Max.].

**Table 2:** Neonatal characteristics and early clinical findings by referral Status.

Variables	Social service referral		*p*
	No (*n* = 97)	Yes (*n* = 96)	
Gestational age^§^	37.0 [25.0–41.0]	37.0 [23.0–41.0]	0.117**
Birth Weight^§^	3,070.0 [620.0–4,490.0]	2,780.0 [560.0–3,800.0]	**0.003** **
Sex^‡^			
Male	60 (61.9)	49 (51.6)	0.197*
Female	37 (38.1)	46 (48.4)	
1-min APGAR score^§^	7.0 [2.0–9.0]	7.0 [2.0–9.0]	0.188**
5-min APGAR score^§^	9.0 [5.0–10.0]	8.0 [4.0–10.0]	**0.001** **
Low Birth Weight^‡^, yes	17 (17.5)	30 (31.6)	**0.036** *
Macrosomia^‡^, yes	2 (2.1)	0 (0.0)	0.497*
IUGR^‡^, yes	14 (14.4)	18 (19.1)	0.497*
Head Circumference^§^	34.0 [22.0–37.0]	34.0 [22.0–37.0]	0.468**
Length^§^	48.0 [32.0–53.0]	49.0 [32.0–55.0]	0.073**
NICU Admission^‡^, yes	91 (93.8)	90 (93.8)	0.999*
NICU Diagnosis^‡^			
No NICU admission	6 (6.2)	4 (4.2)	**0.008** *
Infections	14 (14.4)	9 (9.4)	
Prematurity and related conditions	15 (15.5)	20 (20.8)	
Metabolic disorders	12 (12.4)	4 (4.2)	
Neurological problems	2 (2.1)	14 (14.6)	
Other medical problems	48 (49.5)	45 (46.9)	
Complications of Prematurity^‡^, yes	11 (11.3)	17 (17.7)	0.293*
Respiratory Distress syndrome^‡^, yes	13 (13.4)	19 (19.8)	0.317*
Necrotizing Enterocolitis^‡^			
No	94 (96.9)	85 (88.5)	0.090*
Grade 1	2 (2.1)	5 (5.2)	
Grade 2	1 (1.0)	5 (5.2)	
Grade 3	0 (0.0)	1 (1.0)	
Intraventricular Hemorrhage^‡^, yes	1 (1.0)	3 (3.1)	0.368*
Patent Ductus Arteriosus^‡^, yes	1 (1.0)	5 (5.2)	0.118*
Bronchopulmonary Dysplasia^‡^, yes	0 (0.0)	11 (11.5)	**0.002** *
Cholestasis^‡^	0 (0.0)	1 (1.0)	0.497*
Surfactant administration^‡^, yes	12 (12.4)	19 (19.8)	0.227*
Pneumothorax^‡^, yes	1 (1.0)	5 (5.3)	0.116*
SNAPPE-II Score^§^	4.0 [0.0–46.0]	0.5 [0.0–75.0]	0.770**

APGAR, appearance, pulse, grimace, activity, and respiration score; IUGR, intrauterine growth restriction; NICU, neonatal intensive care unit; SNAPPE-II: score for neonatal acute physiology with perinatal extension-II.

* Pearson Chi-Square, Fisher's Exact/Fisher Freeman Halton test.

** Mann–Whitney *U* test.

‡ *n* (%).

§ Median [Min.–Max.].

Bold values indicate statistically significant results.

**Table 3:** Laboratory results and hospital course by referral Status.

Variables	Social service referral		*p*
	No (*n* = 97)	Yes (*n* = 96)	
TSH^§^	3.4 [0.5–45.0]	2.7 [0.0–16.8]	**0.003** **
FT4^§^	1.8 [1.0–2.5]	1.6 [0.2–2.9]	**<0.001** **
DAT^‡^			
Negative	90 (92.8)	90 (93.8)	0.999*
Positive	7 (7.2)	6 (6.2)	
ROP^‡^			
Mature retina	93 (95.9)	86 (89.6)	0.226*
ROP detected	3 (3.1)	8 (8.3)	
Died before examination	1 (1.0)	2 (2.1)	
ROP Treatment^‡^			
No treatment/Spontaneous regression	96 (99.0)	92 (95.8)	0.254*
Laser Photocoagulation	1 (1.0)	3 (3.1)	
Intravitreal Injection	0 (0.0)	1 (1.0)	
Congenital Anomalies^‡^			
None	90 (92.8)	81 (84.4)	0.709*
Ambiguous Genitalia	1 (1.0)	1 (1.0)	
Minor Anomaly	1 (1.0)	0 (0.0)	
Trisomy 18	0 (0.0)	2 (2.1)	
Congenital Heart Disease	0 (0.0)	2 (2.1)	
Talipes Equinovarus	2 (2.1)	2 (2.1)	
Inguinal hernia	1 (1.0)	1 (1.0)	
Trisomy 21	1 (1.0)	1 (1.0)	
Stüve Wiedemann syndrome	0 (0.0)	1 (1.0)	
Hemangioma	0 (0.0)	2 (2.1)	
Myelomeningocele	0 (0.0)	1 (1.0)	
Cleft Palate	1 (1.0)	1 (1.0)	
Rectal Polyp	0 (0.0)	1 (1.0)	
Age at admission^§^	1.0 [1.0–3.0]	1.0 [1.0–42.0]	0.068**
Respiratory support^‡^, yes	61 (62.9)	54 (56.2)	0.428*
Length of hospital stay (days)^§^	10.0 [2.0–105.0]	15.0 [1.0–488.0]	**<0.001** **
Hearing screning^‡^			
Not performed	9 (9.3)	8 (8.3)	0.999*
Performed	88 (90.7)	88 (91.7)	
Hearing screening^‡^			
Failed in both ears	1 (1.1)	12 (13.6)	**<0.001** *
Failed in one ear	1 (1.1)	10 (11.4)	
Passed in both ears	86 (97.7)	66 (75.0)	
Social-Service Referral Categories^‡^			
Legal/Judicial Reasons	0 (0.0)	20 (20.8)	-
Parental Care and Behavioral Risks	0 (0.0)	40 (41.7)	
Parental Psychosocial and Functional Challenges	0 (0.0)	28 (29.2)	
Socioeconomic/Structural Vulnerabilities	0 (0.0)	8 (8.3)	

DAT, direct antiglobulin test; FT4, free thyroxine (FT4); ROP, retinopathy of prematurity; TSH, thyroid-stimulating hormone.

Statistical significance was defined as *p* < 0.05.

Bold values indicate statistically significant results.

* Pearson Chi-Square, Fisher's Exact/Fisher Freeman Halton test.

** Mann–Whitney *U* test.

‡ *n* (%).

§ Median [Min.–Max.].

**Table 4:** Social-service referral categories and subcategories (*n* = 96).

Category	Subcategory	*n* (%)
Legal/Judicial Reasons (*n* = 20)	Sexual assault of the mother	11 (55.0)
	Paternity testing request	2 (10.0)
	Suspected infant neglect or abuse	1 (5.0)
	Infant born to an incarcerated mother	2 (10.0)
	Identity/documentation problems	2 (10.0)
	Foundling infant*	2 (10.0)
Parental Psychosocial and Functional Challenges (*n* = 28)	Communication or language barriers	16 (57.1)
	Maternal psychiatric disorder	1 (3.6)
	Family refusal of care for a chronically ill infant	2 (7.1)
	Maternal somatic illness	2 (7.1)
	Maternal death during delivery	1 (3.6)
	Disaster-affected family	1 (3.6)
	Uninvolved or uninterested family	5 (17.9)
Socioeconomic/Structural Vulnerabilities (*n* = 8)	Lack of health insurance	5 (62.5)
	Undocumented immigrant family	3 (37.5)
Parental Care and Behavioral Risks (*n* = 40)	Parental substance use	24 (60.0)
	Maternal rejection of the infant	16 (40.0)

* Infant abandoned or without identified parent.

*n* = number of neonates, percentages (%) indicate the proportion within each main category.

**Table 5:** Mortality and discharge outcomes by referral Status.

Variables	Social service referral		*p* *
	No (*n* = 97)	Yes (*n* = 96)	
Mortality^‡^			
Alive	96 (99.0)	89 (92.7)	**0.035**
Exitus	1 (1.0)	7 (7.3)	
Tracheostomy^‡^, yes	0 (0.0)	4 (4.2)	0.059
Discharge status			
Recovered	96 (99.0)	89 (92.7)	**0.035**
Exitus	1 (1.0)	7 (7.3)	
Discharge destination^‡^			
Home	96 (99.0)	36 (37.5)	**<0.001**
Social services placement	0 (0.0)	50 (52.1)	
Exitus	1 (1.0)	7 (7.3)	
Transferred	0 (0.0)	2 (2.1)	
Palliative care center	0 (0.0)	1 (1.0)	

* Pearson Chi-Square, Fisher's Exact/Fisher Freeman Halton test.

‡ *n* (%).

Statistical significance was defined as *p* < 0.05.

Bold values indicate statistically significant results.

**Table 6:** Logistic regression (LR) analysis of factors associated With social -service referral.

Variables	Univariate LR		Multivariate LR	
	OR (95% CI)	*P* value	OR (95% CI)	*P* value
Nationality non Turkish vs. Turkish	4.06 (2.02–8.15)	**<0.001**	1.99 (0.52–7.57)	0.313
Maternal age (years)	0.94 (0.89–0.98)	**0.003**	0.98 (0.9–1.07)	0.682
Legally married: yes vs. no	0.06 (0.03–0.14)	**<0.001**	0.05 (0.01–0.22)	**<0.001**
Antenatal care visit: yes vs. no	0.09 (0.05–0.18)	**<0.001**	0.12 (0.04–0.44)	**0.001**
OGTT performed: yes vs. no	0.25 (0.08–0.78)	**0.017**	0.31 (0.03–3.15)	0.318
Maternal hemoglobin level	0.66 (0.53–0.83)	**<0.001**	0.41 (0.23–0.7)	**0.001**
Mode of delivery: C/S vs. NSD	0.43 (0.23–0.82)	**0.011**	0.39 (0.11–1.47)	0.165
Birth weight (grams)	0.99 (0.98–0.99)	**0.003**	0.99 (0.98–0.99)	0.622
5-min APGAR score	0.58 (0.44–0.78)	**<0.001**	0.69 (0.32–1.49)	0.351
NICU Diagnosis: Ref. =no NICU admission				
Infections	0.96 (0.21–4.39)	0.963	0.22 (0.02–3.13)	0.265
Prematurity & related conditions	1.992 (0.48–8.37)	0.343	0.24 (0.01–4.37)	0.337
Metabolic disorders	0.49 (0.09–2.73)	0.423	0.09 (0.01–2.16)	0.141
Neurological problems	10.5 (1.5–73.68)	**0.018**	4.66 (0.14–154.64)	0.389
Other medical problems	1.41 (0.37–5.31)	0.615	1.01 (0.1–10.37)	0.990
TSH level	0.89 (0.81–0.99)	**0.032**	0.75 (0.59–0.94)	**0.014**
Length of stay (days)	1.04 (1.02–1.07)	**0.001**	1.07 (1.02–1.13)	**0.009**
Hearing test				
Passed bilaterally vs. uni/bilaterally failed	0.07 (0.02–0.31)	**<0.001**	0.28 (0.03–2.39)	0.247

95% CI, %95 confidence interval; APGAR, appearance, pulse, grimace, activity, and respiration score; C/S, cesarean section; NICU, neonatal intensive care unit; NSD, normal spontaneous delivery; OGTT, oral glucose tolerance test; OR, odds ratio; TSH, thyroid-stimulating hormone.

Reference categories: nationality (Turkish), marital status (not married), antenatal care (none), OGTT (no), mode of delivery (NSD), NICU diagnosis (no NICU admission), and hearing test (failed in one or both ears). Bold values indicate statistically significant results (*p* < 0.05).

## Discussion

This analysis offers a comprehensive evaluation of maternal, neonatal, and social determinants associated with social-service referral among infants hospitalized in a tertiary NICU in a resource-limited setting. Rather than reflecting isolated medical complexity, infants referred to social services represent a particularly vulnerable subgroup shaped by cumulative social disadvantage, inadequate antenatal care, and adverse perinatal environments. These intersecting vulnerabilities underline the importance of interpreting neonatal outcomes within a broader biopsychosocial context.

Adolescent pregnancy was substantially more prevalent among referred infants, consistent with prior research demonstrating that adolescent mothers face limited access to healthcare, poor nutrition, incomplete education, and increased social marginalization (14, 15). These structural disadvantages not only compromise maternal and neonatal health but also increase the need for coordinated postnatal social support, thereby contributing to the likelihood of social-service referral.

Lack of antenatal care emerged as another key determinant of social-service referral. Mothers without regular prenatal visits were more likely to require social-service involvement, reflecting a combination of medical, psychosocial, and structural barriers. Previous studies have consistently shown that insufficient antenatal care is associated with adverse pregnancy outcomes and increased perinatal risk among socially disadvantaged populations (16–18). Beyond its clinical role, routine antenatal follow-up facilitates early risk identification, screening for maternal diseases, micronutrient supplementation, and comprehensive perinatal planning, underscoring its protective role.

Maternal substance use was a particularly salient determinant of social-service referral in our cohort, as all substance-exposed infants required social-service notification. This finding underscores the clinical, social, and safety concerns associated with parental addiction in the neonatal period. Substance use is widely recognized as a chronic condition requiring coordinated obstetric, neonatal, primary-care, and social-service support rather than isolated medical management (19, 20). In this context, social-service involvement serves a critical protective function by ensuring infant safety while facilitating linkage of families to ongoing addiction treatment and social-welfare resources.

Marital status also emerged as an important social determinant in relation to social-service referral. Being legally married appeared to confer a protective effect, likely reflecting greater family stability, emotional support, and shared caregiving responsibility. Previous studies similarly indicate that marital partnership is associated with improved maternal well-being, increased healthcare engagement, and more favorable perinatal outcomes, particularly in socially vulnerable populations (21).

Low birth weight among referred infants appears to reflect the cumulative impact of adverse prenatal and social conditions rather than functioning as an isolated trigger for social-service referral. Factors such as inadequate antenatal care, maternal anemia, smoking, and substance use are well-established contributors to impaired fetal growth and frequently coexist in socially vulnerable populations (22, 23). In this context, low birth weight appears to be a consequence of underlying prenatal and social risk factors rather than a direct reason for social-service referral.

Prematurity-related morbidities, including bronchopulmonary dysplasia and neurological complications, appear to reflect the combined effects of biological vulnerability and suboptimal prenatal and perinatal environments. Prenatal stressors, inadequate antenatal surveillance, and perinatal instability have been shown to increase the risk of chronic lung disease and central nervous system injury, particularly among infants born into socially disadvantaged contexts (24, 25). These findings support the notion that adverse neonatal morbidity patterns in referred infants are closely intertwined with broader social and antenatal risk profiles.

Nationality also emerged as an important contextual factor in relation to social-service referral. While non-Turkish nationality was associated with referral in univariate analysis, this association did not persist after multivariable adjustment, suggesting that nationality itself was not an independent determinant. Rather, migrant status likely functioned as a proxy for coexisting vulnerabilities, including inadequate antenatal care, unmarried status, language barriers, and limited integration into the healthcare system. Similar findings have been reported in studies from high-income countries, where adverse perinatal outcomes among migrant families are primarily driven by structural disadvantage rather than ethnicity or nationality alone (18, 26).

Hearing-screen outcomes among referred infants likely reflect the combined influence of biological vulnerability and social disadvantage. Established clinical risk factors such as NICU admission, mechanical ventilation, and low birth weight are known to adversely affect auditory outcomes (27). In addition, social factors—including reduced caregiver engagement, unmarried motherhood, language barriers, and challenges in healthcare navigation—may further compromise timely follow-up and completion of outpatient hearing assessments (28, 29). Prolonged hospitalization in this population may also amplify these risks by increasing cumulative exposure to medical interventions and delaying continuity of post-discharge care.

Importantly, social-service involvement in our center is not conceptualized as a separation-based or automatic intervention. Decisions regarding mother–infant separation are made on a case-by-case basis and are guided primarily by the underlying reason for social-service notification. As an initial step, family-centered interviews are conducted focusing on the specific social concern prompting referral, with the aim of evaluating family capacity, needs, and available support systems. When indicated, home visits are planned according to the nature of the social concern to allow contextual assessment of the caregiving environment. The final decision regarding separation, when considered necessary, is therefore informed by the outcomes of these family interviews and home evaluations rather than by the referral itself. In line with contemporary family-centered care principles, mother–infant separation is not routinely employed as a first-line approach following social-service referral.

However, due to the retrospective design of this study, detailed information regarding the timing of referrals, parental involvement in decision-making, home visit findings, and specific post-referral social interventions was not consistently available, limiting direct assessment of their impact on attachment preservation and family coping.

An additional important finding of this study is the presence of social risk factors among neonates in the non-referral group. This observation suggests that social vulnerability may not always be fully recognized in the absence of overt clinical severity or clearly documented social indicators during NICU hospitalization. In settings where social-service referral relies largely on clinical judgment rather than standardized screening, some families with significant social risk may remain unidentified. This under-recognition underscores the need for structured and systematic social-risk assessment tools integrated into routine NICU practice, which may facilitate earlier identification of vulnerable families and ensure more equitable access to supportive social-service interventions.

Discharge to institutional care among infants referred to social services appears to reflect broader concerns related to caregiving capacity, psychosocial instability, and child safety, rather than isolated neonatal clinical factors. This interpretation is consistent with international literature indicating that protective placement is often considered when family resources or caregiving environments are deemed insufficient to meet the infant's needs (30). Similarly, the increased vulnerability observed in this population likely represents the cumulative impact of adverse antenatal and social conditions, including limited prenatal care, adolescent motherhood, maternal substance use, and broader social instability, as reported in previous studies (31, 32).

Overall, these findings underscore the importance of integrated perinatal–social care strategies and the early identification of social risk during pregnancy and neonatal hospitalization. Infants requiring social-service involvement constitute a biologically and socially fragile population, and timely, family-centered supportive interventions may help mitigate adverse outcomes while preserving attachment whenever possible.

## Conclusion

This study highlights that social-service referral in the NICU reflects a convergence of medical vulnerability and adverse social determinants rather than isolated clinical factors. Understanding social-service involvement as a supportive, family-centered process underscores the importance of early identification of social risk during pregnancy and neonatal hospitalization. Integrating structured social-risk assessment into perinatal and neonatal care may help ensure timely, equitable, and supportive interventions for vulnerable families, particularly in resource-limited settings.

### Strengths

This study examines maternal social determinants and neonatal outcomes within a tertiary NICU using systematically collected clinical data. While prior reviews have explored broad social vulnerabilities in migrant or underserved populations, few studies have evaluated their clinical implications in high-acuity neonatal environments (18, 33). A major strength of this study is the structured classification of referral reasons and the integrated analysis of their associations with maternal and neonatal variables. The predominance of familial and legal reasons among referral categories highlights that social-service involvement extends beyond economic disadvantage to encompass caregiving capacity, safety concerns, and family dynamics. Additionally, the findings raise new research questions regarding antenatal social-risk screening and long-term neurodevelopmental outcomes among referred infants.

### Limitations

This study has several limitations inherent to its retrospective design. Detailed information regarding the timing of social-service referrals, parental involvement in decision-making, home visit findings, and specific post-referral social interventions was not consistently available, precluding direct assessment of their impact on attachment preservation and family coping. In addition, social-service referral in routine NICU practice may be influenced by clinical judgment rather than standardized social-risk screening, raising the possibility of under-recognition of social vulnerability among some non-referred families. Finally, as this was a single-center study, the generalizability of the findings to other healthcare settings may be limited.

## Data Availability

The original contributions presented in the study are included in the article/Supplementary Material, further inquiries can be directed to the corresponding author.
